# Multi-SNP Haplotypes in Circadian *PER3* Gene Are Associated with Mood and Sleep Disorders in University Students

**DOI:** 10.3390/genes16091047

**Published:** 2025-09-05

**Authors:** Francesca Goodell, Krista K. Ingram

**Affiliations:** Department of Biology, Colgate University, Hamilton, NY 13346, USA

**Keywords:** mood disorders, sleep disorders, anxiety, depression, circadian clock, chronotype, PER3, internalizing disorders, mental health

## Abstract

**Background**: Mood disorders, including anxiety, depression, and seasonal affective disorder (SAD), are often comorbid and can be exacerbated by the misalignment of an individual’s circadian rhythm with their social timing. Single-nucleotide polymorphisms (SNPs) in circadian clock genes have been associated with both internalizing disorders and sleep disturbances, and some clock polymorphisms, including those in the *Period3 (PER3)* gene, likely function via delaying or advancing circadian period and affecting sleep–wake patterns. **Methods**: Here, we explore associations of multiple SNP haplotypes in the *PER3* gene with anxiety, depression, internalizing disorder (ID), chronotype, and sleep disturbance in young adults (*n* = 1109 individuals). **Results**: We report novel, sex-specific associations of single *PER3* SNPs with mood and sleep disorders and highlight strong multi-SNP haplotype associations, revealing a greater risk of mood and sleep disorders in university students with specific *PER3* haplotypes. **Conclusions**: Our results suggest that the additive effects of multiple risk variants amplify the prevalence of mood disorders and sleep disruptions in young adults. Understanding how polymorphisms within circadian genes interact to alter clock function, sleep-wake behavior and downstream physiological changes in the brain may help explain the comorbidity of mood and sleep syndromes and provide future therapeutic targets to combat these debilitating disorders.

## 1. Introduction

Internalizing disorders, including anxiety and depression, are becoming increasingly prevalent worldwide [1], with around one in five adults suffering from mental health related problems, resulting in a reduced quality of life for many individuals [2,3]. Mood disorders are often co-morbid with sleep disruptions, particularly in young adults, suggesting similar molecular and physiological pathways regulate symptoms of these common disorders. One potential link involves the influence of circadian rhythms and social timing on mental health, with numerous studies detailing the influence of social jetlag and chronotype on mood and sleep disorders e.g., [4,5,6,7].

Circadian rhythms are regulated by an endogenous, 24-h molecular clock that is influenced by both internal and external factors. In humans, this clock is controlled by a cluster of cells located in the suprachiasmatic nucleus (SCN), which is responsible for a person’s sleep–wake cycle and related downstream physiological changes [8]. Clock cells regulate cyclical protein expression via a series of transcriptional–translational feedback loops. The dysregulation of clock gene expression patterns is associated with mental health disorders, including anxiety, depression, and seasonal affective disorder (SAD) [9,10,11,12,13,14]. Moreover, genome-wide association (GWAS) and candidate gene studies have provided evidence that circadian clock genes are associated with chronotype/diurnal preference, sleep disturbance, and mood disorders [15,16,17,18,19,20,21,22,23]. Growing evidence also suggests that the evening-type chronotype, characterized by a delay in circadian phase, can be a risk factor for major depressive disorder (MDD), SAD, anxiety disorders, and poorer sleep quality [7,24,25,26]. In many individuals, mood and sleep disorders are comorbid. For example, individuals with anxiety tend to experience sleep disturbances and a greater reliance on sleep medications, while those with depression face amplified daytime dysfunction [27]. The comorbidity of sleep and mood disorders suggests the likelihood of shared molecular and physiological mechanisms influencing their symptoms.

*PER3*, a homologue of clock genes *PER1* and *PER2,* is considered a non-essential clock gene, as knockouts of the *PER3* gene in rodents alter only the phase, but not the function, of circadian rhythms. Large GWASs on chronotype and mood/sleep disorders have failed to directly implicate PER3 [28], but numerous candidate gene studies have found associations between polymorphisms in the PER3 gene and anxiety (e.g., [29]), depression (e.g., [10,23,30]), seasonal affective disorders (e.g., [15,31]), and sleep disorders (e.g., [15,16,32,33]), suggesting that there may be a threshold effect requiring multiple synergistic interactions between variants. *PER3* has a stabilizing effect on *PER1* and *PER2* proteins [29], suggesting that the transcription of *PER3* plays an important role in regulating one’s sleep and mood amidst sleep–wake cycle mismatches and seasonal changes in circadian rhythms [30].

This study examines the associations between four *PER3* SNPs and anxiety, depression, SAD, internalizing disorder (ID), and sleep disturbances. Individual SNPs were chosen based on previously reported associations with one or more mood or sleep disorders. Our aims were to determine if these single SNP associations with mood disorders and sleep disturbance in the *PER3* gene were also prevalent in our young adult population, whether the associations are sex-specific, and which variants predict the presence of an internalizing disorder (in this study, ID designation includes symptoms of anxiety, depression, and/or SAD). In addition, we investigate whether specific *PER3* SNP haplotypes increase or decrease the risk of mood or sleep disorders in young adults, indicating additive effects of multiple variants within this gene influence mood and sleep regulation pathways.

## 2. Materials and Methods

### 2.1. Participants

Data was collected as part of a multi-year study of Colgate University students and their circadian rhythms and mood (total sample: *n* = 1109; median age 19.21). Participants were primarily recruited using consecutive sampling from introductory biology courses composed mostly of first- and second- year undergraduates from the years 2013 to 2024. Eligibility criteria included only a requirement for age (18 years or older). We did not screen for diagnoses of psychiatric or substance abuse disorders or family history of mental illness. All collection methods were approved by the Institutional Review Board at Colgate University (#FR-F13-07, #ER-F14-12, #F15-13, #ER-F16-19, #ER-S24-05, #ER-F25-06), and all participants gave written informed consent prior to the start of this study. All data were re-coded for confidentiality, and access to data was limited to the PI and research technician. DNA collections (hair) were destroyed within 24 months of collection.

### 2.2. Self-Reported Surveys

In addition to providing a hair sample, each participant was asked to complete a computer-based survey including the reduced (5-question) Horne–Östberg Morningness–Eveningness Questionnaire (rMEQ [34]), the Beck Depression Inventory (BDI-II [35]), the trait version of the Spielberger’s State–Trait Anxiety Scale (STAI [36]), the Seasonal Pattern Assessment Questionnaire (SPAQ [37]), and the short form of the Patient-Reported Outcomes Measurement Information System Sleep Disturbance (PROMIS™ [38]).

The results of the rMEQ characterize diurnal preference with a score of 4–7 being definitely-evening, 8–11 being moderately evening, 12–17 being neither type, 18–21 being moderately morning, and 22–25 being definitely morning. The BDI-II is a diagnostic test for depression with scores that range from 0 to 60. Individuals with scores < 14 are categorized as not depressed, 14–19 are mildly depressed, 20–28 are moderately depressed, and >28 are severely depressed. STAI scores ranging from 20 to 80 are used to gauge anxiety. Scores between 20 and 37 indicate no or low anxiety, between 38 and 44 moderate anxiety, and between 45 and 80 high anxiety. The SPAQ is a self-reported measure of seasonality and SAD. SPAQ scores can range from 0 to 24, with scores ≥ 12 indicating the presence of SAD. The PROMIS sleep disturbances test (SLEEP) measures self-reported sleep disturbance and ranges from 8 to 40. Scores < 25 signify no or slight sleep disturbance, 26–29 mild disturbances, 30–37 moderate disturbances, and 38+ severe sleep disturbances. Finally, the presence of an internalizing disorder was characterized by a BDI ≥ 14 and/or an STAI score ≥ 38 and/or an SPAQ score ≥ 12.

### 2.3. Genotyping

DNA was extracted from the 10 to 20 hair follicles provided by each participant. The follicles were then digested for 24 h at 56 °C with Proteinase K, after which these samples were further purified using the DNAeasy Micro Kit (Qiagen, Frederick, MD, USA). Genotyping for *PER3A*, *PER3B*, and *PER3C* was conducted using TaqMan SNP Genotyping assays; SNPs were identified using real-time qPCR protocols. The VNTR length polymorphism in exon 18 of the *PER3* gene was analyzed using a SeqStudio Genetic Analyzer. Detailed information about each SNP is provided in Table 1. PCR was performed using a Qiagen PCR Master Mix (Qiagen, MD, USA) in 25 μL volumes. A negative water control was included in each plate of PCR samples. The PCR cycling conditions included 3 min. at 94 °C, followed by 35 cycles of 45 s at 94 °C, 45 s at 58 °C, and 45 s at 72 °C, and a final run at 72 °C for 3 min. *PER3* VNTR alleles were separated by capillary electrophoresis and sized using the GeneScan Rox 500 Size Standard (Life Technologies, Carlsbad, CA, USA).

### 2.4. Statistical Analysis

Associations of *PER3* VNTR, *PER3A*, *PER3B*, and *PER3C* were individually tested with scores from the STAI, BDI, ID, SLEEP, and rMEQ surveys. Odds ratios were calculated for each *PER3* SNP for the entire population and, separately, for males and females. One-way tests of analysis of variance (ANOVAs) were also performed for separate survey scores. Chi-square independence tests were performed on the numbers of individuals with an internalizing disorder (ID) of each genotype, with additional analyses separately performed for males and females. All statistical calculations were performed using SPSS (IBM SPSS v 29).

For our multi-SNP analysis, we had complete *PER3* haplotype data for 508 individuals, encompassing 23 different multi-SNP haplotypes. We initially performed t-tests on the 16 most common haplotypes, comparing each haplotype’s average STAI, BDI, PROMIS sleep scores and ID percentage against the averages for the overall sample. Based on these results, the six haplotypes that significantly differed for mood variables and the four that significantly differed for sleep disturbances were chosen for further analyses. Odds ratios were calculated for associations between multi-SNP haplotypes and STAI, BDI, ID, and PROMIS sleep disturbance. One-way ANOVAs were used to determine significant survey score differences between multi-SNP haplotype groups.

Generative artificial intelligence (GenAI) has not been used in this study design or paper preparation.

## 3. Results

### 3.1. Results from Single SNP Analyses

Odds ratios were used to test for associations of the risk allele for each *PER3* SNP (the 4-repeat allele of *PER3* VNTR, the G-allele of *PER3A*, the A-allele of *PER3B*, and the T-allele of *PER3C* with anxiety, depression, and ID (Table 2); for associations with sleep disturbances and evening chronotype (Table 3); and for sex-specific associations between sleep or mood disorders and *PER3* SNPs (Table 4).

The VNTR 4-repeat allele is associated with an increased risk for having an internalizing disorder (OR = 2.22, 95% CI: 1.1240 to 4.3973, *p* = 0.0217) in the whole sample population and in males (OR = 3.07, 95% CI: 1.0777 to 8.7544, *p* = 0.0357). Individuals without ID are more frequent within the 5/5 genotype for both the overall sample (X^2^ = 6.381; df = 2; *p* = 0.041) and, marginally, in males only (X^2^ = 4.822; df = 2; *p* = 0.090). However, there was no significant difference found in the percentage of females with an ID across VNTR genotypes (X^2^ (2, *n* = 489) = 2.406, *p* = 0.300).

**Figure 1:**
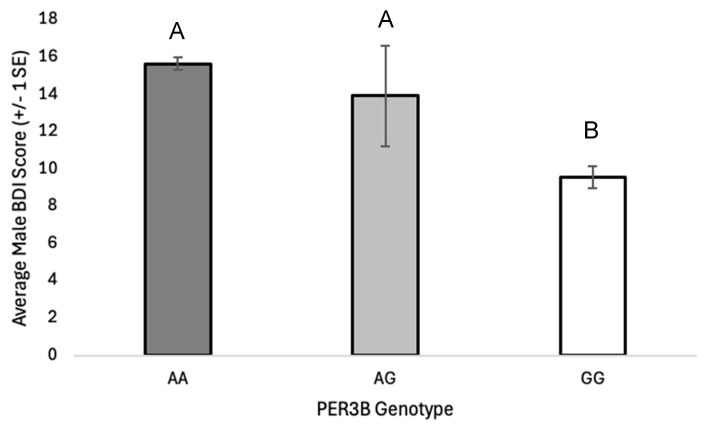
Average male BDI scores (±1 SE) in the total sample with *PER3B* genotypes AA, AG, and GG. Genotype had a significant effect on male BDI score (F_2, 172_ = 3.381, *p* = 0.036, η^2^ = 0.04). Homogenous subsets (A, B) determined by post-hoc Tukey test, *α* = 0.05.

**Figure 2:**
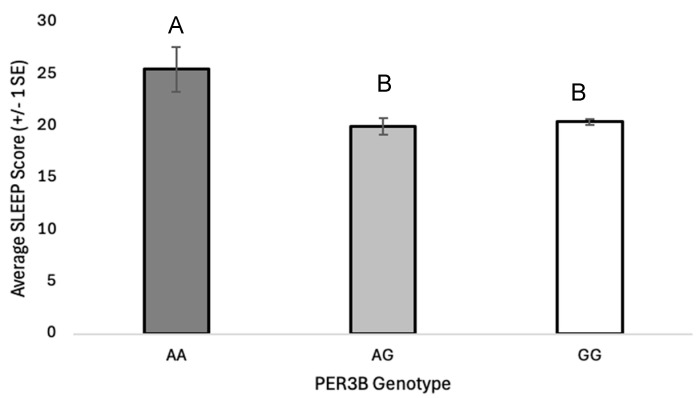
Average PROMIS sleep scores (±1 SE) in the total sample with *PER3B* genotypes AA, AG, and GG. Genotype had a significant effect on sleep score (F_2, 596_ = 2.674, *p* = 0.070, η^2^ = 0.02). Homogenous subsets (A, B) determined by post hoc Tukey test, *α* = 0.05.

The *PER3B* A-allele is associated with an increased risk of depression (OR = 1.69, 95% CI: 1.0356–2.7498, *p* = 0.0357). This risk appears to be marginally greater in males with at least one A-allele (OR = 2.42, 95% CI: 0.9546 to 6.1503, *p* = 0.0626). The average BDI score is lower in *PER3B* GG male homozygotes than *PER3B* AA and *PER3B* AG males (Figure 1; Table S1; F_2, 172_ = 3.381, *p* = 0.036). The average PROMIS sleep score is marginally greater in *PER3B* AA individuals than *PER3B* AG or *PER3B* GG individuals (Figure 2; Table S1; F_2, 596_ = 2.674, *p* = 0.070). The *PER3B* A-allele is also associated with an increased risk of having an evening-chronotype (OR = 1.65, 95% CI: 1.0053 to 2.7155, *p* = 0.0476).

### 3.2. Associations from Multi-SNP Analyses

Odds ratios were calculated for the six most significant multi-SNP *PER3* haplotypes (Table 5) in relation to anxiety, depression, and ID (Table 6) and the four most significant multi-SNP *PER3* haplotypes in relation to sleep disturbances (Table 7).

Multi-SNP haplotype 6 shows the largest effect in our study; individuals with this haplotype have over eight times the risk of depression relative to other haplotypes (OR = 8.45, 95% CI: 1.00 to 72.9003, *p* = 0.0502), although this result is marginally significant with a low sample size of six individuals. Individuals with haplotype 1 have nearly three times higher risk of anxiety (OR = 2.80, 95% CI: 1.2433 to 6.2847, *p* = 0.0129) and twice the risk of an internalizing disorder (OR= 2.38, 95% CI: 0.9140 to 6.1276, *p* = 0.0725). Multi-SNP haplotypes 3 and 4 are significantly associated with a two-fold decrease in the risk of an internalizing disorder (haplotype 3: OR = 0.43, 95% CI: 0.2006 to 0.9259, *p* = 0.0310; haplotype 4: OR = 0.36, 95% CI: 0.1338 to 0.9577, *p* = 0.0407).

Multi-SNP haplotype 2 is associated with a three-fold decrease in the risk of sleep disturbances (OR = 0.36, 95% CI: 0.1567 to 0.8226, *p* = 0.0154), while haplotype 5 is marginally associated with a three-fold increase in the risk of sleep disturbances (OR= 2.64, 95% CI: 0.9397 to 7.4387, *p* = 0.0654). Finally, multi-SNP haplotypes 7 and 8 are marginally associated with a seven-fold risk of sleep disturbance (haplotype 7: OR = 6.82, 95% CI: 0.7032 to 66.1462, *p* = 0.0977; haplotype 8: OR = 6.82, 95% CI: 0.7032 to 66.1462, *p* = 0.0977), with only four individuals within each haplotype group.

**Figure 3:**
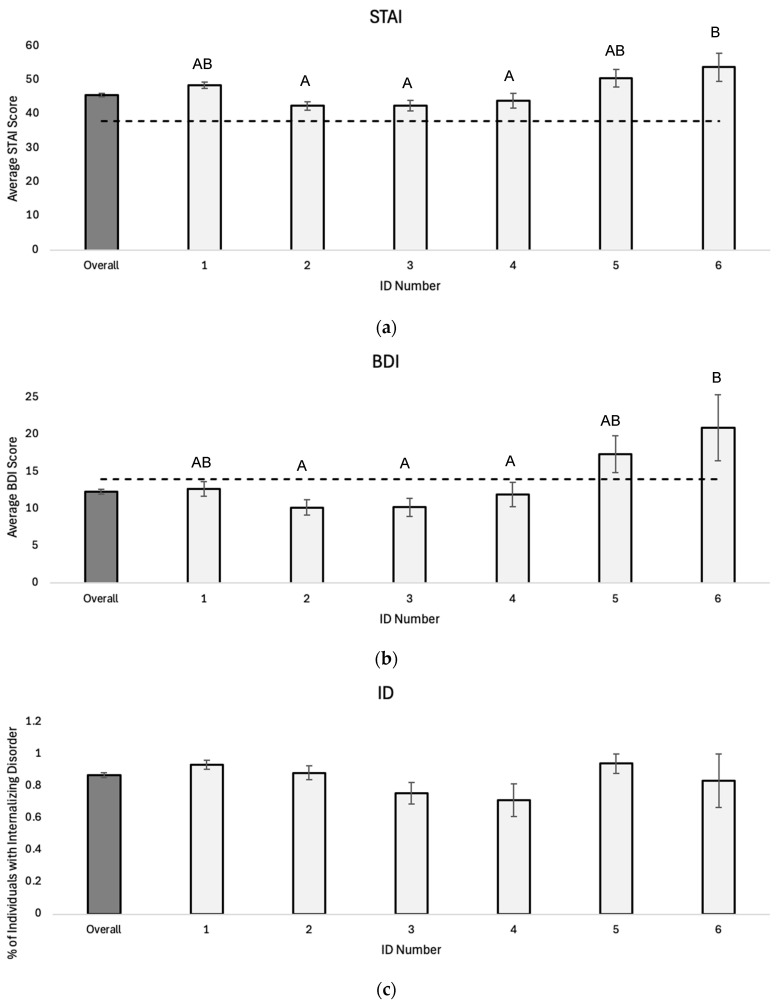
(**a**) Average STAI score (±1 SE), (**b**) average BDI score (± 1 SE), and (**c**) percentage of individuals with an internalizing disorder (±1 SE) for multi-SNP *PER3* haplotypes 1–6. Multi-SNP haplotype had a significant effect on average STAI score (F_5, 207_ = 5.496, *p* < 0.01), average BDI score (F_5, 205_ = 4.178, *p* < 0.01), and percentage of individuals with an internalizing disorder (F_5, 207_ = 2.547, *p* = 0.03). Homogenous subsets determined by post hoc Tukey test, *α* = 0.05. Shaded bar represents population average for total sample (*n* = 508). Dashed line represents threshold score for diagnosis of disorder.

**Figure 4:**
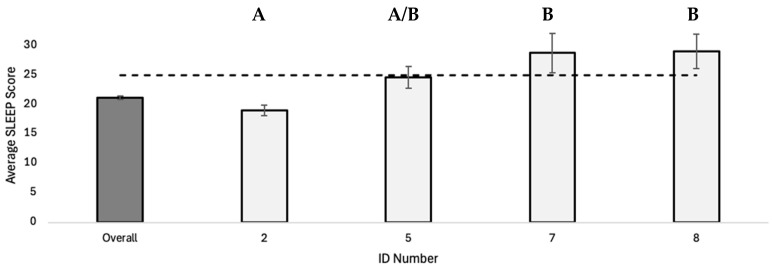
Average PROMIS sleep score (±1 SE) for multi-SNP haplotypes 2, 5, 7 and 8. Multi-SNP *PER3* haplotype had a significant effect on average sleep score (F_3, 66_ = 7.175, *p* < 0.01). Homogenous subsets (A, B) determined by post hoc Tukey test, *α* = 0.05. Shaded bar represents population average for total sample (*n* = 508). Dashed line represents threshold score for diagnosis of disorder.

Comparisons across multi-SNP haplotypes revealed that average STAI, BDI, and PROMIS sleep scores, as well as the percentage of individuals with ID, significantly differ by multi-SNP *PER3* haplotype (Figure 3 and Figure 4; STAI: F_5, 207_ = 5.496, *p* < 0.01; BDI: F_5, 205_ = 4.178, *p* < 0.01; ID: F_5, 207_ = 2.547, *p* = 0.03; SLEEP: F_3, 66_ = 7.175, *p* < 0.01). Post hoc Tukey tests also reveal a significant difference in multi-SNP haplotypes 2, 3, and 4 with multi-SNP haplotype 6 for average STAI score (Figure 3a) and average BDI score (Figure 3b). In addition, there is a significant difference in average PROMIS sleep score between haplotype 2 and haplotypes 7 and 8 (Figure 4; F_3, 66_ = 7.175, *p* < 0.01).

## 4. Discussion

Previous studies have linked single *PER3* variants with both mood and sleep disorders, suggesting that the *PER3* gene may play a role in modulating mental health and sleep quality. In this study, we report multiple associations of single PER3 SNPs with anxiety, depression, sleep disturbance, and internalizing disorder in a population of young adults. In addition, we find stronger associations between multi-SNP *PER3* haplotypes and these disorders, suggesting that the additive effects of clock gene variants may have significant impacts on mood and sleep quality in young adults.

### 4.1. Association of Single SNP Genotypes with Mood Disorders and Sleep Disturbances

The *PER3* variable number tandem repeats (VNTR) SNP (rs57875989) is a 54-nucleotide unit that is repeated four or five times within exon 18 of the *PER3* gene. Previous studies have reported associations between the shorter length VNTR allele (four repeats) and evening chronotype [12,17,29] as well as anxiety and depression [29,30]. In this study, we found that individuals with at least one VNTR 4-repeat allele have twice the risk of reporting an internalizing disorder than 5/5 homozygous individuals, and males possessing a VNTR 4-repeat had three times the odds of having anxiety than GG homozygous males. In addition, the percentage of individuals and males with an internalizing disorder significantly differed between *PER3* VNTR genotypes. Few studies have examined gene associations with generalized ID as an outcome variable, but this result is consistent with smaller studies by Liberman and colleagues [29,30] which noted the 4-repeat allele was associated with increased odds of both anxiety and depression in young adults.

The *PER3A* (rs228697) polymorphism is associated with a C-to-G base change resulting in a proline-to-alanine substitution at amino acid position 864. The *PER3A* SNP has been found to be associated with both diurnal preference and mood disorders, with the G-allele more frequent in evening types and GG homozygotes more likely to report evening preferences [18,29,30]. In addition, one study reports an additive allelic effect of the G-allele with anxiety with individuals with two G-alleles reporting higher anxiety levels than individuals with one G-allele [29]. Shi and colleagues [23] also found an association between the G-allele of *PER3A* and depression. Interestingly, we observed no single SNP association of *PER3A* with any of the tested disorders in this study. This may be due to a low frequency of the risk allele, G, in our study population, or it may suggest this polymorphism has a weaker association with mood or sleep disorders that develop in younger individuals.

The *PER3B* SNP (rs17031614) is associated with a relatively rare G-to-A base change with the frequency of the A-allele of approximately 0.04. However, unlike *PER3A* and *PER3C*, this base change does not alter the amino acid sequence, maintaining a serine at position 872. The *PER3B* variant has not been studied as frequently as other clock polymorphisms, and results have varied by population. A previous study found the A-allele to be associated with depression [23], while GG homozygosity has been found to be more common in females suffering from anxiety [24] and/or sleep disturbances [33]. Our study also found numerous associations between *PER3B* and depression, the presence of an internalizing disorder, evening chronotype, and sleep disturbances with *PER3B*. Individuals with at least one *PER3B* A-allele had nearly twice the odds of having depression than GG homozygotes, with males having greater than twice, which is consistent with results of Shi and colleagues [23]. Further analysis showed a statistically significant difference in average male BDI score according to genotype, with individuals possessing an A-allele having higher BDI scores. We also found that individuals possessing a *PER3B* A-allele had nearly twice the odds of having an evening chronotype compared to GG homozygotes. This result has not been explicitly noted in other studies; however, it is not unexpected, given the fact that evening chronotypes are frequently associated with depression. Interestingly, average PROMIS sleep scores significantly differed between the three *PER3B* genotypes, indicating that the presence of a G-allele may be protective against sleep disturbances.

Lastly, the *PER3C* polymorphism (rs10462020) is associated with a T-to-G base change resulting in a valine-to-glycine substitution at amino acid position 674. Studies have found an association between the *PER3C* SNP and diurnal preference, with the G-allele being associated with the morning chronotype [31] and the T-allele with sleep disturbances, particularly Delayed Sleep Phase Syndrome (DSPS; [32]). In this study, we uncovered a novel, sex-specific association, as males possessing a *PER3C* T-allele had four times the risk of reporting anxiety compared to those who were GG homozygotes. This result is consistent with previous findings that the G-allele is associated with morning-types, the chronotype that generally reports decreased levels of anxiety and depression. However, because our sample size for males was relatively small in this study, this should be considered a preliminary finding, requiring replication in a larger study with balanced cohorts

### 4.2. Association of Multi-SNP Haplotypes with Mood Disorders and Sleep Disturbances

One of the most interesting results from this study is the variance in mood and sleep scores across different multi-SNP *PER3* haplotypes. For example, haplotype 1 increased an individual’s risk of both anxiety and ID. These individuals are homozygous for VNTR, *PER3B*, and *PER3C* risk alleles for anxiety and/or depression and/or an evening chronotype, so this haplotype association is consistent with the single SNP expectations. On the other hand, multi-SNP haplotypes 3 and 4 are associated with lower ID risk, even though both haplotypes contain genotypes that are homozygous for risk alleles previously found to be associated with anxiety, depression, or evening chronotype. However, individuals with haplotypes 3 and 4 also have protective genotypes for the other PER3 SNPs which may mitigate the effects of the risk alleles. Finally, individuals with haplotype 6 have an increased risk of having depression. This haplotype is particularly interesting as participants with haplotype 6 possess only one of the potential eight risk alleles previously found to be associated with depression, the *PER3C* T-allele.

Our *PER3* haplotype analysis also reveals several associations with sleep quality. Multi-SNP haplotypes 5, 7, and 8 were all found to elevate the risk of sleep disturbances relative to the population average, and individuals with haplotype 2 reported lower sleep disturbance scores. Haplotype 8 individuals are homozygous for the *PER3B* and *PER3C* risk alleles known to be associated with sleep disturbances. However, haplotypes 5 and 7 show similar effects on sleep scores but contain fewer risk alleles than haplotype 8.

Of the eight multi-SNP haplotypes evaluated, haplotypes 1–4 reported fewer depression symptoms with average scores less than 14 on the BDI, while average BDI scores for haplotypes 5 and 6 showed an increased risk of depression symptoms in young adults. A similar pattern can be seen for average PROMIS sleep scores; individuals with haplotype 2 have lower average PROMIS sleep scores, while haplotypes 7 and 8 report higher-than-average sleep scores. Across mood and sleep disorders, haplotype 2 appears to be a protective factor for both sleep disturbance and depression, while haplotype 5 appears to be a risk factor for sleep disturbance, depression, anxiety, and internalizing disorder. It is important to note that haplotypes 6, 7, and 8 contained less than 10 individuals in the current study, which is not unexpected given the low frequencies of the risk alleles for two of the four SNPs evaluated in this study. Although it is difficult to determine the robustness of the associations reported in our sample, these preliminary results provide a compelling direction for future exploration of these *PER3* polymorphisms in larger population samples.

Unfortunately, our results for multi-SNP haplotype analysis for anxiety and ID were less conclusive than for depression and sleep disturbance. This is likely due to the fact that average STAI scores (45.7) in our young adult population were above the cutoff value used to determine the presence of anxiety (≥38), indicating our participants are more anxious, on average, than the general population. When looking at the percentage of individuals in this study with an internalizing disorder, we face a similar challenge. Approximately 87% of our sample reported an internalizing disorder, rendering the effects of haplotypic differences harder to discern in this population.

Although the direct role of PER3 in the etiology of mood and sleep disorders is not yet known, it is thought that post-transcriptional modifications and ligand interactions involving PER3 interfere with the phosphorylation of PER1 and PER2—mediating the stability and turnover of these core circadian clock proteins and altering the period length and coordination of circadian rhythms [30,41,42]. In addition, PER3 is likely to be involved in mechanisms critical for the rapid adaption to changing environmental conditions, e.g., conditions related to shift work [43]. Thus, the additive effects of multiple polymorphisms in PER3 may increase the likelihood of circadian disruption, leading to altered rhythms which then modulate downstream regulatory processes that influence mood and sleep homeostasis (reviewed in [7,26,44]). The synergistic interactions of circadian disruption and unstable social environments, like those found in residential university systems, may act to amplify circadian misalignment and exacerbate mood and sleep disorders in these populations.

### 4.3. Limitations

One limitation of this study is our reliance on self-reported survey data regarding the presence or absence of mood disorders and/or sleep disturbances. Future studies using clinical diagnostic interviews or actigraphy or PSG to objectively measure sleep could strengthen conclusions. Eligibility for this study did not include criteria excluding individuals with diagnosed psychiatric or substance use disorders or a family history of mental illness, which may have impacted study outcomes. Due to the lack of diversity in the sample population (participants were primarily young, Caucasian adults of European descent), our results may not be representative of the general population. Haplotype or allelic associations may differ in older populations or populations with diverse ethnic backgrounds. For example, future studies should address if the *PER3* associations found in university students are age-related or whether they are found in other populations of individuals experiencing chronic social jetlag and thus are more generally associated with circadian misalignment. In addition, *PER3* and other circadian clock variants display different allele frequencies across diverse populations, suggesting that particular variants may influence mood and sleep disorders in some populations but not others. This cross-population allelic variability is another rich area for future research in understanding how redundancies in the circadian clock mechanism alter the influence of particular variants on downstream processes involved in mental and sleep health.

In our methods for calculating odds ratios for *PER3* associations, we did not employ a correction for multiple testing because we tested few, planned comparisons for each disorder. Thus, there is a risk of false positives in our results. Some haplotype groups are represented by only a few individuals, resulting in low power for these analyses. In addition, we have not replicated these results in independent cohorts. The robustness of the reported associations should be considered preliminary findings that may be strengthened by testing with larger sample sizes in additional populations. Finally, the average anxiety scores in our study participants appear to be higher than the general population and likely represent a selection bias due to the competitive environment of introductory science courses and/or increased academic stress at selective liberal arts colleges. The high anxiety scores may indicate a ceiling effect in this study for identifying haplotype associations related to anxiety and, potentially, ID.

## 5. Conclusions

Our study reports multiple associations of *PER3* SNPs with mood and sleep disorders in university students. In addition, *PER3* haplotype analysis reveals strong associations with anxiety, depression, ID, and sleep disturbances. Our results indicate that additive effects of risk alleles in the *PER3* gene may influence mood disorders and sleep quality. Haplotype analyses can detect specific variant combinations associated with the comorbidity of mood and sleep disturbances and may provide clues to identifying the molecular mechanisms that regulate these disorders, offering common therapeutic targets to combat these debilitating disorders.

## Figures and Tables

**Table 1:** Descriptive information about the four Period 3 SNPs. The studies referenced are not an exhaustive list.

SNPs	Alleles	Global Frequencies (Study Population)	Examples of Studies Showing Associated Risks
*PER3* VNTR rs57875989	4 5	5—0.30–0.40 (0.274)	4—evening chronotype [12,17,29,39], anxiety, and depression [29,30]
*PER3A* rs228697	C G	G—0.10 (0.096)	G—evening chronotype [18,29,30], anxiety [30], and depression [23]
*PER3B* rs17031614	G A	A—0.04 (0.071)	A—depression [23] GG—anxiety [24] and sleep disturbances [33] in females
*PER3C* rs10462020	T G	G—0.17 (0.180)	T—evening chronotype [31,40] and sleep disturbances [32]

**Table 2:** Odds ratio values for anxiety, depression, and internalizing disorders for each of the four *PER3* SNPs ordered from greatest risk (gray shading) to most protective (white shading). *p*-values < 0.05 are denoted by ** and rows in bold.

Association of Risk Allele with	SNPs	n	Odds Ratio	95% CI	Z-Score	*p*-Value
**STAI**	PER3C	654	1.6609	0.7409 to 3.7233	1.232	0.2180
	PER3A	970	1.2031	0.8512 to 1.7004	1.047	0.2950
	PERB	646	1.0768	0.6504 to 1.7830	0.288	0.7735
	VNTR	921	0.9487	0.5725 to 1.5721	0.204	0.8381
**BDI**	**PER3B**	**623**	**1.6875**	**1.0356 to 2.7498**	**2.100**	0.0357 **
	PER3C	632	1.4380	0.5620 to 3.6791	0.758	0.4486
	VNTR	667	0.7684	0.4248 to 1.3899	0.871	0.3837
	PER3A	677	0.7302	0.4672 to 1.1411	1.381	0.1674
**Internalizing** **Disorder (ID)**	**VNTR**	**706**	**2.2232**	**1.1240 to 4.3973**	**2.296**	0.0217 **
	PER3A	735	1.5351	0.7680 to 3.0682	1.213	0.2251
	PER3C	560	1.2263	0.3468 to 4.3367	0.317	0.7516
	PER3B	552	0.6724	0.3485 to 1.2974	1.184	0.2366

**Table 3:** Odds ratio values for sleep disturbances and evening chronotype for each of the four *PER3* SNPs, ordered from greatest risk (gray shading) to most protective (white shading). *p*-values < 0.05 are denoted by ** and rows in bold.

Association of Risk Allele with	SNPs	n	Odds Ratio	95% CI	Z-Score	*p*-Value
**Sleep Disturbance**	PER3C	605	1.7547	0.5878 to 5.2381	1.008	0.3136
	PER3A	914	1.1044	0.7465 to 1.6340	0.497	0.6192
	VNTR	866	1.0444	0.5741 to 1.9002	0.142	0.8867
	PER3B	599	0.9877	0.5716 to 1.7069	0.044	0.9648
**rMEQ-ET**	**PER3B**	**606**	**1.6523**	**1.0053 to 2.7155**	**1.981**	0.0476 **
	PER3C	614	1.6281	0.6401 to 4.1412	1.023	0.3062
	VNTR	881	0.9549	0.5698 to 1.6003	0.175	0.8610
	PER3A	929	0.7668	0.5297 to 1.1100	1.407	0.1594

**Table 4:** Odds ratio values for select sex-specific associations between sleep or mood and *PER3* SNPs ordered from greatest risk to lowest risk (risk values shaded in gray). *P* -values < 0.1 are denoted by *, and *p*-values < 0.05 are denoted by ** and rows in bold.

Association of Risk Allele with	SNPs	n	Odds Ratio	95% CI	Z-Score	*p*-Value
**M STAI**	PER3C	181	4.0909	0.7715 to 21.6925	1.655	0.0979 *
	PER3B	180	1.9130	0.7108 to 5.1490	1.284	0.1991
**M BDI**	**PER3B**	**175**	**2.4231**	**0.9546 to 6.1503**	**1.862**	0.0626 *
**M ID**	PER3C	153	4.1379	0.5592 to 30.6219	1.391	0.1643
	**VNTR**	**216**	**3.0714**	**1.0776 to 8.7544**	**2.100**	0.0357 **
**F BDI**	PER3B	448	1.5007	0.8441 to 2.6681	1.383	0.1667
**F SLEEP**	PER3C	439	3.2267	0.7308 to 14.2467	1.546	0.1221

**Table 5:** Overview of identification numbers used for each multi-SNP *PER3* haplotype.

Haplotype ID Number	n	VNTR	PER3A	PER3B	PER3C
1	76	4/4	CC	GG	TT
2	52	4/5	CC	GG	TG
3	41	5/5	CC	GG	TT
4	21	4/4	CC	AG	TT
5	17	4/4	CG	GG	TG
6	6	5/5	CC	GG	TG
7	4	4/4	CC	AA	TT
8	4	4/4	GG	GG	TT

**Table 6:** Odds ratio values for anxiety, depression and internalizing disorders for six select multi-SNP *PER3* haplotypes ordered from greatest risk (gray shading) to most protective (white shading). † indicates haplotype group size < 10. *p*-values < 0.1 are denoted by “*”; *p*-values < 0.05 are denoted by “**”.

Association of Haplotype with	Haplotype ID	Haplotype n, Overall N	Odds Ratio	95% CI	Z-Score	*p*-Value
STAI	5	17, 506	4.1649	0.5458 to 31.7806	1.376	0.1688
	**1**	**76, 506**	**2.7953**	**1.2433 to 6.2847**	**2.487**	0.0129 **
	6 †	6, 506	1.2657	0.1462 to 10.9544	0.214	0.8306
	2	52, 506	0.6526	0.3388 to 1.2570	1.276	0.2019
	3	41, 506	0.5800	0.2849 to 1.1809	1.502	0.1332
	4	21, 506	0.4872	0.1913 to 1.2405	1.508	0.1315
BDI	**6†**	**6, 501**	**8.4511**	**1.0001 to 72.9003**	**1.941**	0.0502 *
	5	17, 501	1.4880	0.5641 to 3.9254	0.803	0.4219
	1	76, 501	1.3233	0.8070 to 2.1701	1.110	0.2669
	4	21, 501	1.2500	0.5165 to 3.0251	0.495	0.6207
	3	41, 501	0.6877	0.3409 to 1.3873	1.046	0.2957
	2	52, 501	0.6609	0.3515 to 1.2428	1.285	0.1987
ID	5	17, 508	2.4847	0.3241 to 19.0497	0.876	0.3811
	1	76, 508	2.3795	0.9140 to 6.1276	1.796	0.0725 *
	2	52, 508	1.1840	0.4850 to 2.8902	0.371	0.7107
	6†	6, 508	0.7569	0.0871 to 6.5803	0.252	0.8007
	**3**	**41, 508**	**0.4310**	**0.2006 to 0.9259**	**2.157**	0.0310 **
	**4**	**21, 508**	**0.3580**	**0.1338 to 0.9577**	**2.046**	0.0407 **

**Table 7:** Odds ratio values for sleep disturbances for four select multi-SNP *PER3* haplotypes ordered from greatest risk (gray shading) to most protective (white shading). † indicates haplotype group size <10. *p*-values < 0.1 are denoted by “*”, and *p*-values < 0.05 are denoted by “**”.

Association of Haplotype with	Haplotype ID Number	n	Odds Ratio	95% CI	Z-Score	*p*-Value
sleep	7 †	4, 459	6.8201	0.7032 to 66.1462	1.656	0.0977 *
	8 †	4, 459	6.8201	0.7032 to 66.1462	1.656	0.0977 *
	5	17, 459	2.6439	0.9397 to 7.4387	1.842	0.0654 *
	**2**	**52, 459**	**0.3591**	**0.1567 to 0.8226**	**2.422**	0.0154 **

## Data Availability

The original contributions presented in this study are included in the article/Supplementary Material. Further inquiries can be directed to the corresponding author.

## Supplementary Materials

The following supporting information can be downloaded at: https://www.mdpi.com/article/10.3390/genes16091047/s1, Table S1. Raw data for genotypes and haplotypes.

## Abbreviations

The following abbreviations are used in this manuscript:

PER3	Period 3 gene
SNP	Single-nucleotide polymorphism
VNTR	Variable number of tandem repeats
SAD	Seasonal affective disorder
ID	Internalizing disorder
MDD	Major depressive disorder
ET	Evening chronotype
